# Adherence to and Persistence with Adalimumab Therapy among Swedish Patients with Crohn’s Disease

**DOI:** 10.3390/pharmacy10040087

**Published:** 2022-07-20

**Authors:** Yifei Liu, Joakim Söderberg, Jingdong Chao

**Affiliations:** 1 Division of Pharmacy Practice and Administration, The University of Missouri–Kansas City School of Pharmacy, Kansas City, MO 64108, USA; 2 Health Solutions, 118 26 Stockholm, Sweden; joakim.soderberg@healthsolutions.se; 3 Regeneron Pharmaceuticals, Inc., Tarrytown, NY 10591, USA; jingdong.chao@regeneron.com

**Keywords:** medication adherence, medication persistence, health locus of control, adalimumab

## Abstract

Objectives: (1) to determine the adherence and persistence rates of adalimumab therapy among Swedish patients with Crohn’s disease (CD), and (2) to compare self-administration devices to predict the medication adherence and persistence. Methods: We conducted a retrospective analysis of the Swedish National Board of Health and Welfare database during a unique time period, when both the pen and the syringe were available. The pen was proposed to indicate a larger extent of internal control, according to health locus of control. Medication adherence was defined as a medication possession ratio (MPR) ≥ 0.8. A patient was considered nonpersistent if the time between any two dispensing records, minus the days of supply dispensed exceeded 180 days. The predictors of adherence were evaluated using a logistic regression, and the predictors of persistence were evaluated using a Cox proportional hazards model. Results: Among the 1083 patients studied, 89% were adherent and 77% were persistent. The patients using the pen and the patients treated in gastroenterology centers were more likely to be adherent and less likely to be nonpersistent. Conclusions: The adherence rate to adalimumab therapy was 89% and the one-year persistence rate was 70%. The pen and treatment in a gastroenterology center had a positive impact on the adherence and persistence among Swedish patients with CD.

## 1. Introduction

Crohn’s disease (CD), a common type of inflammatory bowel disease (IBD), has become a global disease [1]. In Europe, the direct health care costs are approximately EUR 3500 for CD per patient per year [2]. Additionally, there is a clear north–south gradient for CD incidence rates, with European countries in the north having a much higher rate [3]. In Sweden, the CD incidence rate was 8.1 per 100,000 person–years from 1990 to 2014 [4], and the prevalence of CD was 0.19% in 2010 [5].

Medication adherence and persistence have important implications for the long-term management of CD, because the ultimate goal of therapy is to induce and maintain remission. Medication adherence is “the extent to which a patient acts in accordance with the prescribed interval and dose of a dosing regimen,” whereas medication persistence is “the duration of time from initiation to discontinuation of therapy” [6].

Health locus of control distinguishes the dimensions of internal and external control [7,8]. The former is the belief that health is under one’s own control, whereas the latter is the belief that health is under the control of external factors. Health locus of control has an impact on medication adherence [9,10]. In general, internal control is associated with better medication adherence [9], although occasionally opposite findings are demonstrated [10]. Nevertheless, in terms of predicting health outcomes, while plenty of studies have compared the impact of internal vs. external control factors, fewer have examined the varying extent of control within the same dimension of either internal or external control.

For patients with moderate to severe CD who do not respond to glucocorticoids or immunomodulators, tumor necrosis factor (TNF) inhibitors including adalimumab therapy, are recommended to induce and maintain remission [11,12]. There was a time period in Sweden when two types of self-administration devices for adalimumab therapy were available at the same time. One was a single-use, prefilled auto-injection pen, and the other was a single-use, prefilled glass syringe. Compared with the syringe, the pen was relatively new and had an improved design enclosing a syringe with protective caps and an activator button [13].

According to health locus of control, the self-administration of either device would be under a patient’s internal control. Yet, with its improved design to help patients carry and administer the medication, the pen would indicate a larger extent of internal control than the syringe. We hypothesized that a larger extent of internal control would yield better health outcomes. Namely, the pen would lead to better adherence to and persistence with adalimumab therapy than the syringe. The objectives of our study were to (1) determine the adherence and persistence rates of adalimumab therapy among Swedish patients with CD, and (2) compare the pen vs. the syringe to predict the medication adherence and persistence in this population.

## 2. Methods

This was a retrospective cohort study of adalimumab dispensing records, among Swedish patients with CD. We focused on the time period from 6 July 2005 to 30 September 2009, because of the availability of 2 self-administration devices in Sweden during this period, and also because of data access. The Swedish National Board of Health and Welfare is a government agency that maintains data in the areas of health, medical care, and social services [14]. Study subjects were all patients with CD in the Swedish National Board of Health and Welfare database. The dispensing records contained the following information: patient age group, sex, days of supply associated with a dispensing activity, self-administration device (pen or syringe), prescriber specialty (gastroenterologist or other), and prescriber practice setting (gastroenterology, internal medicine, or other).

**Figure 1:**
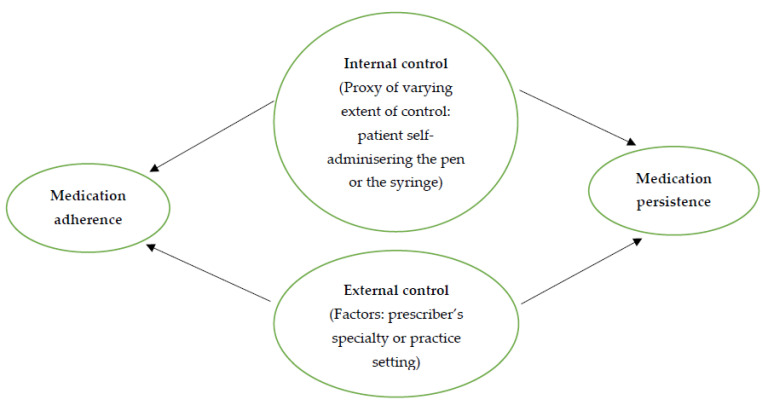
Proposed framework, based on health locus of control and data available.

Utilizing health locus of control, as mentioned earlier, we proposed that the self-administration device would be a proxy of the varying extent of internal control (Figure 1), and the pen would be associated with a larger extent. In addition, we proposed that prescriber characteristics such as specialty and practice setting would be factors of external control. Furthermore, the health outcomes would include both medication adherence and persistence. Of note, owing to secondary data, we were unable to directly measure the variables of health locus of control with survey items. Instead, we used the self-administration device as a proxy for the varying extent of internal control, and prescriber characteristics as factors of external control.

The index date was the date of the first adalimumab dispensing record. If a patient did not switch self-administration device or prescriber (either specialty or practice setting), the censoring date was 30 September 2009. If a patient switched the device or prescriber, the refill date before the earliest switch date was the censoring date. That is, for patients who made a switch, we only examined the dispensing records before the switch.

Refill adherence is one way to capture medication adherence and can be measured by medication possession ratio (MPR) [15]. We calculated MPR for each patient using the days of supply dispensed (excluding the last dispensing record), divided by the number of days between the first and last dispensing records, from the index date to the censoring date. Patients who had only 1 dispensing record were excluded from MPR calculation. Then patients were identified as adherent (MPR ≥ 0.8) vs. non-adherent (MPR < 0.8).

To capture persistence, we measured the duration of therapy for each patient by the time gap between any 2 dispensing records, minus the days of supply dispensed, with the earlier record from the index date to censoring date. For patients who had 1 dispensing record, the duration of therapy was measured by the time gap between the dispensing record and the censoring date, minus the days of supply dispensed. In Sweden, the reimbursement regulations only allowed the dispensing of medications for up to a 90-day supply at a time and a patient could not refill the prescriptions until 60 days after the dispensing. Therefore, a patient was considered non-persistent if the time gap was greater than 180 days.

Descriptive statistical analyses were performed for adherence (yes/no), MPR, persistence (yes/no), days of persistence, and the other variables. A logistic regression was conducted, in which the outcome variable was medication refill adherence (yes/no) and the independent variables included age group, sex, dispensing device, prescriber specialty, and prescriber practice setting. A Cox proportional hazards model was developed, in which the outcome variable was medication non-persistence (yes/no), and the independent variables were the same as in the logistic regression. We used the Statistical Analysis System (SAS Institute, Cary, NC, USA) to restructure the original data and the Statistical Package for Social Science (IBM Corporation, Armonk, NY, USA) to analyze the data after the data restructure.

## 3. Results

A total of 1083 patients were identified and included in the analyses, of which 52.6% were female and 61.3% used the pen (Table 1). The prescriber for most patients was a gastroenterologist, and more than half of the patients had a prescriber who worked in an internal medicine setting. The average MPR was 0.93, with 790 patients (89%) identified as medication adherent (MPR ≥ 0.8) and 837 patients (77%) identified as medication persistent.

The self-administration device and the prescriber practice setting were significant predictors of medication adherence in the logistic regression (Table 2). Compared with the patients using a syringe, the patients using a pen were more likely to be adherent, with an odds ratio (OR) of 1.78 (95% confidence interval [CI]: 1.14, 2.78). The patients whose prescribers worked in a gastroenterology center were more likely to be adherent than the patients whose prescribers worked in an internal medicine center, with an OR of 1.70 (95% CI: 1.04, 2.79).

In the Cox proportional hazards model of medication non-persistence, the self-administration device, the prescriber practice setting, and the sex of the patient were significant predictors (Table 3). The patients using a pen were less likely to be nonpersistent than those using a syringe, with a hazard ratio (HR) of 0.74 (95% CI: 0.56, 0.97). The patients whose prescribers worked in a gastroenterology center were less likely to be nonpersistent than the patients whose prescribers worked in an internal medicine center, with an HR of 0.64 (95% CI: 0.48, 0.86). Compared with the female patients, the male patients were less likely to be nonpersistent, with an HR of 0.75 (95% CI: 0.57, 0.97).

## 4. Discussion

In this retrospective cohort study, the medication adherence rate was 89%. It was consistent with a pooled adalimumab adherence rate of 83% in a systematic review of adalimumab-treated patients with IBD [16]. Knowledge of the factors associated with TNF inhibitor adherence and persistence may help health care professionals to identify patients at risk of nonadherence and non-persistence, and to modify their prescribing practices or their medication management strategies among Swedish patients with CD.

Our study shows that health locus of control helps understand medication adherence and persistence. The predictive utility of the pen supported our hypothesis. Based on the improved design of the pen [13], we hypothesized that the self-administration of the pen would give patients a larger extent of internal control than the syringe. In fact, the pen is perceived as easier to use and more convenient [17]. Moreover, patients experience less pain when using the pen as opposed to the syringe, to self-administer adalimumab therapy [18]. The literature suggests that internal control is generally associated with better medication adherence than external control [9]. What this study adds to the literature, is that within the dimension of internal control, a larger extent of control is associated with better adherence to and persistence with adalimumab therapy. Therefore, the different extent of internal control can play a role in determining health outcomes.

Between the two external factors, the prescriber’s practice setting affected both the medication adherence and persistence, whereas the prescriber’s specialty had no impact. Specifically, the patients treated in a gastroenterology center were 70% more likely to be adherent to adalimumab therapy and 36% more likely to be persistent with adalimumab therapy, compared with the patients treated in an internal medicine center. There may be various aspects of medication management in a specialty practice setting that positively influence patients’ adherence and persistence, such as enhanced patient education and prescriber communication. Also, patients who visit a gastroenterology center may have more severe conditions, and thus may be more adherent to and persistent with the therapy.

In our analysis, the male patients with CD were less likely to be nonpersistent with adalimumab therapy than the female patients. This finding has been previously reported, though the results across the studies are inconsistent. Kane and Dixon reported that female patients with CD were less likely to be adherent to infliximab therapy [19], and Liu et al. found that women with CD were less adherent to adalimumab therapy than men [20]. However, sex has not predicted medication adherence in other studies of patients with IBD [16,21,22,23].

Our study had four limitations. First, the 0.8 MPR threshold used to define adherence is commonly used in medication adherence research, but whether the threshold is clinically optimal for a specific study sample is unknown [24]. Additionally, we assumed that medication refill was equivalent to medication usage. Second, because we retrospectively extracted dispensing-related information from a secondary database, we did not have any information on the baseline disease characteristics, such as the disease activity, disease duration, concomitant and prior therapies, the duration of therapy, comorbidities, surgical history, or other disease- and treatment-related factors that may have influenced the patients’ medication adherence and persistence. Third, our study had limited generalizability. Our analysis included only Swedish patients with CD and may not be generalizable to other non-northern European populations. Similarly, we only included adalimumab therapy, and the results may not be generalizable to other therapies. Fourth, the study timeframe was constrained by data access. Unfortunately, we do not have access to updated data.

For future research, updated or more specific data are needed to understand the reasons underlying TNF inhibitor adherence and persistence among patients with CD. For example, further explorations of disease-related factors may allow clinicians to target patients with appropriate disease management strategies. In addition, understanding the specific factors that explain the better medication adherence and persistence in gastroenterology centers may allow health care systems to optimize their treatment paradigms for patients with CD.

## 5. Conclusions

In this study, among Swedish patients with CD, the medication adherence rate was 89% and the one-year medication persistence rate was 70%. Under health locus of control, the self-administration device of adalimumab therapy could be regarded as a proxy for varying internal control. In Swedish patients with CD, the pen (a proxy for a larger extent of internal control than the syringe) and treatment in a gastroenterology center (an external control factor) had a positive impact on both the adherence to and persistence with adalimumab therapy.

## Figures and Tables

**Table 1:** Patient and prescriber characteristics.

Characteristic	Values
Adherence, *n* (%)	
Yes (MPR ≥ 0.8)	790 (88.6)
No (MPR < 0.8)	102 (11.4)
Total ^a^	892 (100)
MPR	
Mean (SD)	93.47 (14.97)
Range	9.98–100
Total, N ^a^	892
Persistence, *n* (%)	
Yes (gap ≤ 180 days)	837 (77.3)
No (gap > 180 days)	246 (22.7)
Total	1083 (100)
Days of persistence	
Mean (SD)	233.09 (217.12)
Range	0–1315
Total, N	1083
Age group, *n* (%)	
20–29	300 (29.0)
30–39	246 (23.8)
40–49	237 (22.9)
50+	251 (24.3)
Total, N ^a^	1034 (100)
Sex, *n* (%)	
Women	570 (52.6)
Men	513 (47.4)
Total	1083 (100)
Self-administration device, *n* (%)	
Pen	664 (61.3)
Syringe	419 (38.7)
Total	1083 (100)
Prescriber specialty, *n* (%)	
Gastroenterology	994 (96.2)
Others	39 (3.8)
Total ^b^	1033
Prescriber practice setting, *n* (%)	
Internal medicine center	598 (55.3)
Gastroenterology center	413 (38.2)
Others	71 (6.5)
Total ^b^	1082

^a^ Total numbers vary because of missing value and exclusion of cases for which MPR was not calculable. ^b^ Total numbers vary because of missing values. MPR = medication possession ratio; SD=standard deviation.

**Table 2:** Logistic regression model of medication possession ratio (N ^a^ = 818).

Independent Variables	Beta Coefficient	Odds Ratio (95% Confidence Interval)
Age group ^b^		
30–39	−0.40	0.67 (0.37, 1.21)
40–49	−0.29	0.75 (0.41, 1.36)
50+	0.30	1.36 (0.69, 2.65)
Sex (male) ^c^	−0.06	0.94 (0.61, 1.46)
Self-administration device (pen) ^d^	0.58 ^e^	1.78 (1.14, 2.78)
Prescriber specialty (other) ^f^	0.66	1.94 (0.44, 8.54)
Prescriber practice setting ^g^		
Gastroenterology	0.53 ^e^	1.70 (1.04, 2.79)
Other	0.93	2.53 (0.76, 8.42)

^a^ N = 818 due to missing values. ^b^ The reference group is 20–29 years. ^c^ The reference group is female. ^d^ The reference group is syringe. ^e^ Significant at the 0.05 level. ^f^ The reference group is gastroenterology. ^g^ The reference group is internal medicine.

**Table 3:** Cox proportional hazards model of adalimumab non-persistence.

**Regression Model**	**Adalimumab Non-Persistence (N ^a^ =961)**	
Chi-square	20.48 ^b^	
**Independent Variables**	**Beta Coefficient**	**Hazard Ratio** **(95% Confidence Interval)**
Age group ^c^		
30–39	−0.05	0.95 (0.65, 1.38)
40–49	−0.10	0.90 (0.62, 1.32)
50+	0.11	1.11 (0.78, 1.59)
Sex (Men) ^d^	−0.30 ^e^	0.75 (0.57, 0.97)
Dispensing device (pen) ^f^	−0.30 ^e^	0.74 (0.56, 0.97)
Prescriber specialty (other) ^g^	−0.02	0.99 (0.46, 2.11)
Prescriber practice setting ^h^		
Gastroenterology	−0.45 ^b^	0.64 (0.48, 0.86)
Other	0.08	1.08 (0.59, 2.00)

^a^ N = 961 due to missing values. ^b^ Significant at the 0.01 level. ^c^ The reference group is 20–29 years. ^d^ The reference group is female. ^e^ Significant at the 0.05 level. ^f^ The reference group is syringe. ^g^ The reference group is gastroenterology. ^h^ The reference group is internal medicine.

## Data Availability

Data sharing is not applicable to this article.
